# Theoretical Study of Intramolecular Interactions in Peri-Substituted Naphthalenes: Chalcogen and Hydrogen Bonds

**DOI:** 10.3390/molecules22020227

**Published:** 2017-02-02

**Authors:** Goar Sánchez–Sanz, Ibon Alkorta, José Elguero

**Affiliations:** 1 Irish Centre of High-End Computing, Grand Canal Quay, Dublin 2, Ireland; 2 Instituto de Química Médica, CSIC, Juan de la Cierva, 3, E–28006 Madrid, Spain; iqmbe17@iqm.csic.es

**Keywords:** chalcogen bonds, non-covalent interactions, MP2, interaction energy, intramolecular interactions, hydrogen bonds

## Abstract

A theoretical study of the peri interactions, both intramolecular hydrogen (HB) and chalcogen bonds (YB), in 1-hydroxy-8YH-naphthalene, 1,4-dihydroxy-5,8-di-YH-naphthalene, and 1,5-dihydroxy-4,8-di-YH-naphthalene, with Y = O, S, and Se was carried out. The systems with a OH:Y hydrogen bond are the most stable ones followed by those with a chalcogen O:Y interaction, those with a YH:O hydrogen bond (Y = S and Se) being the least stable ones. The electron density values at the hydrogen bond critical points indicate that they have partial covalent character. Natural Bond Orbital (NBO) analysis shows stabilization due to the charge transfer between lone pair orbitals towards empty Y–H that correlate with the interatomic distances. The electron density shift maps and non-covalent indexes in the different systems are consistent with the relative strength of the interactions. The structures found on the CSD were used to compare the experimental and calculated results.

## 1. Introduction

Non-covalent interactions, especially hydrogen bonds (HBs), are known to be responsible for the conformation and 3D structure of biomolecules like proteins and DNA. Other non-covalent interactions as halogen bonds [1,2,3], pnicogen bonds [4,5] and tetrel bonds [6,7] can contribute as well to the stability of certain molecular conformations. The study of intramolecular interactions is very important in the design of pharmaceutical drugs, particularly in the context of conformationally flexible molecules. Conformation-controlling intramolecular interactions in drug molecules have a direct influence on the binding modes of the drugs with the respective targets [8,9,10]. In particular, intramolecular peri-interactions have been widely studied in the literature in naphthalene and other related systems [11,12,13,14,15].

Chalcogen bonds [16,17,18,19,20,21] (YB) are one of the less studied non-covalent interactions. However, a few articles have been devoted in the literature to the study of intramolecular YBs. For instance, Sanz et al. studied the chalcogen-chalcogen interactions in β-chalcogenovinylaldehydes [22,23] and its corresponding saturated derivatives [24]; Iwaoka et al. studied the nature of the Se···O interactions in benzeneselenenyl derivatives by means of ^17^O-NMR [25]; Nzibo and Scheiner studied the influence of S···O chalcogen bonds in substituted phenyl-SF_3_ molecules [26]; Shishkin and coll. explored the S···O interaction in the X-ray structure of thioindirubin [27]; Mikherdov analyzed the influence of chalcogen bonds in the regio-isomerization of Pd(II) complexes [28]; experimental studies involving S–H···S and Se–H···Se in thiophenol and selenophenol were carried out by Guru Row et al. [29]; and we studied the chalcogen-chalcogen interaction in 2,2′-bifuran, 2,2′-bithiophene, and 2,2′-bitellurophene derivatives [30]. The intramolecular interaction of organoselenium derivatives has been reviewed by Mukherjee et al. [31].

The nature of the YB has been rationalized by Politzer et al. based on the σ–hole concept [32,33,34]. The term σ–hole refers to the electron-deficient outer lobe of a p orbital involved in forming a covalent bond. Therefore, in YBs, as well as in other non-covalent interactions, the importance of the electrostatic interaction term is uppermost [35,36,37,38,39,40,41,42,43,44,45,46]. Several works have been carried out regarding the tunability of the mentioned σ–holes in different types of interactions, including halogen, chalcogen, and pnicogen [47,48,49,50]. In particular, the effect on the σ–hole upon substitution on aromatic rings has been previously studied [47,51,52,53]. However, in the present manuscript, we focus our attention on the simplest cases of intramolecular interactions with no additional substituents in the naphthalene rings. In forthcoming publications the effect of substituents on the aromatic ring on different weak interactions will be considered.

**Scheme 1:**
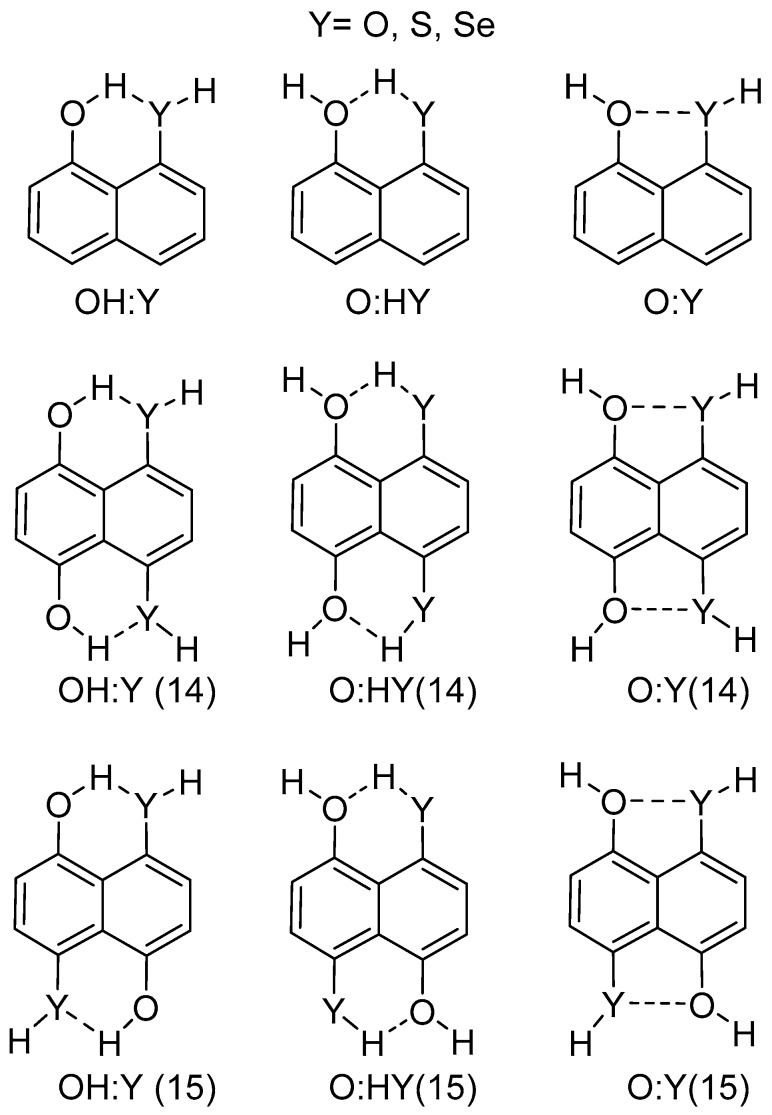
Schematic description of the systems considered; 14 and 15 labels refer to the position of oxygen (OH) atoms.

In the present article, we explore the competition between intramolecular HB and YB in 1-hydroxy-8YH-naphthalene, 1,4-dihydroxy-5,8-di-YH-naphthalene, and 1,5-dihydroxy-4,8-di-YH-naphthalene. For each derivative, two potential HB complexes and one stabilized by YB have been considered (Scheme 1). The first line of Scheme 1 represents the conformations tested for the systems with a single interaction intramolecular hydrogen bond (IMHB^1^) and intramolecular chalcogen bond (IMYB^1^) while the second and third line correspond to those systems with two simultaneous interactions. For simplicity, only the symmetric structures with two identical interactions have been chosen (IMHB^2^ and IMYB^2^). In order to study those structures quantum chemical calculations at the MP2 level, natural bond orbital (NBO), atoms in molecules (AIM), and electron density shift maps have been used.

## 2. Results

### 2.1. Structure and Energy of IMHB^1^ and IMYB^1^ Compounds

**Figure 1:**
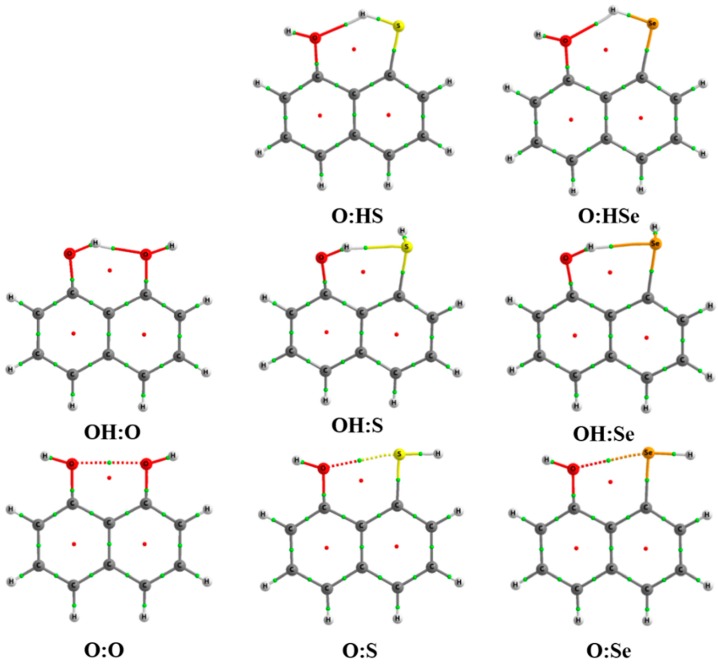
Molecular graph for all the compounds with a single interaction at the MP2/aug-cc-pVDZ computational level. Green and red dots correspond to bond and ring critical points, respectively.

A total of eight systems involving a single interaction have been found. Of those, five correspond to intramolecular hydrogen bonds (IMHB^1^) and three to intramolecular chalcogen bonds (IMYB^1^). Molecular graphs of each system are depicted in Figure 1. Three of them involve HBs in which the oxygen moiety (OH) acts as hydrogen donor, while in the other two the chalcogen (S and Se) atom is the hydrogen donor.

Intramolecular distances obtained in all the cases are shorter than the sum of the van der Waals radii of the atoms involved (vdW_OH_ = 2.72 Å, vdW_SH_ = 3.0 Å, vdW_SeH_ = 3.1 Å, vdW_OO_ = 3.04 Å, vdW_OS_ = 3.32 Å, and vdW_OSe_ = 3.42 Å) [54]. The calculated Y–H···Y′ angles are within the range of the expected HBs, being those relatively more acute when S and Se are the hydrogen donors (Table 1). In the case of chalcogen interactions, the O···Y′–H angles are closer to 180° than in the HB cases. Finally, in all the systems considered with a single interaction, the C–Y···Y′–C is 0°, indicating that all the atoms involved in the intramolecular interaction are within the molecular plane defined by the naphthalene backbone.

The molecular electrostatic potential (MEP) on the 0.001 a.u. electron density isosurface was obtained for the three fragments, which contain only one YH group and plotted in Figure S1. As observed, the maximum value associated to the hydrogen atom bonded to the Y atom varies 0.0823 (O) > 0.0358 (S) > 0.0278 (Se), according to the electronegative nature of the Y atom. These values indicate, as expected, that O is a better hydrogen donor than S and Se. Regarding the σ-hole, no maximum value was found for the OH group. However, the σ-hole is deeper (more positive) in the Se derivative than in the S derivative, consistent with the polarizability of the heteroatom considered.

The relative stability of each compound was studied at the MP2/CBS level and the corresponding relative energies gathered in Table 2. The system with two hydroxyl groups shows only a single minimum with one of the groups acting as hydrogen bond donor and the other as acceptor. In the case of sulfur derivatives, OH:S is the most stable compound in which the oxygen atom acts as hydrogen donor, followed by O:S, and finally O:HS in which the sulfur atom is acting as HB donor. A similar range of relative energies was found in the selenium compounds compared to the sulfur derivatives. However, the relative energies in sulfur derivatives are 0.0 < 10.5 < 14.9 kJ·mol^−1^ for OH:S, O:S, and O:HS respectively while in the selenium compounds they are 0.0 < 8.8 < 21.9 kJ·mol^−1^ for OH:Se, O:Se, and O:HSe indicating that the chalcogen interaction in the Se derivative is stronger than in S derivatives, which is coherent with the electronegativity and polarizability of the chalcogen atoms.

**Scheme 2:**
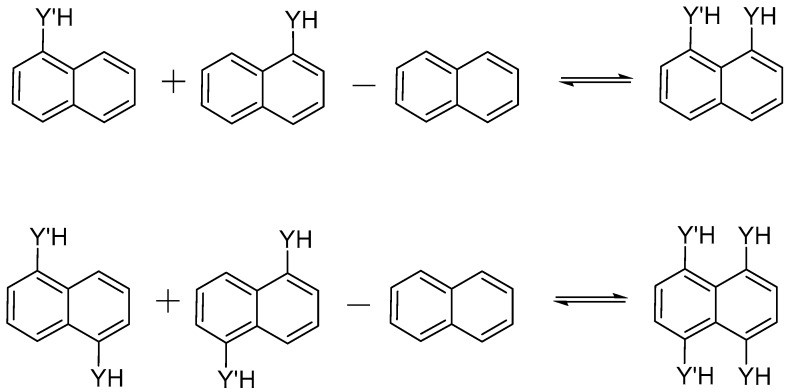
Isodesmic reaction used to obtain the interaction energy.

Regarding the intramolecular interaction energies, different terms were taken into account in order to address such interactions. In the first place, interaction energies, E_int_, were considered through the partition of Scheme 2, keeping the structure of each fragment fixed in the complex geometry, i.e., in the structure with the pertinent interaction. Additionally, the isodesmic energy, E_iso_, was also obtained using the same Scheme 2 but each fragment was relaxed and minimized. Finally, the deformation energy, E_def_, was calculated as the difference between E_iso_ − E_int_, and accounts for the reorganization energy of each fragment.

As observed, compounds bonded by IMHB in which the O is acting as HB donor show negative E_int_ indicating an attractive interaction. However, the rest of the interactions present positive E_int_. The most stable system is OH:O (−12.6 kJ·mol^−1^) followed by OH:Se (−12.3 kJ·mol^−1^). In the case of chalcogen interactions, the E_int_ evolves with the acceptor capacity of the chalcogen, O:O > O:S > O:Se. Regarding the E_iso_, only one compound shows negative isodesmic energy, OH:O (−3.6 kJ·mol^−1^), while the rest are positive. OH:S and OH:Se present small positive E_iso_, while the rest of the compounds exhibit E_iso_ ranging from 12.5 to 25.5 kJ·mol^−1^ the latter being associated to O:HSe compound. As observed for E_int_, IMHB compounds with O as donor are the most stable. Surprisingly, the deformation energies are lower in the IMYB systems O:S and O:Se (5.4 and 7.2 kJ·mol^−1^ respectively) than in the IMHB compounds. The largest reorganization energies of all the compounds studied are found for the O:HS and O:HSe systems, with a penalty of 14.9 and 19.5 kJ·mol^−1^ respectively. The positive values of E_int_ and E_iso_ in conjunction with the large deformation energies indicate that those IMHB in O:HY are unlikely to occur. 

The O:HSe structure presents an imaginary frequency (118 cm^−1^) which corresponds to a rotation around the C–Se axis (no symmetry constrains were imposed). This is a transition state which connects with the OH:Se minimum. However, for the sake of comparison this structure was also considered.

Finally, despite that the interaction energies were obtained at the MP2/CBS, we have evaluated the performance of the basis set calculation E_int_, E_iso_, and E_def_ values at the MP2/aug-cc-pVQZ. Energies values found were very similar to those for MP2/aug-cc-pVTZ and MP2/CBS, which indicates the convergence of the calculations in terms of a basis set (Table S1).

### 2.2. Structure and Energy of IMHB^2^ and IMYB^2^ Compounds

Once the compounds with a single interaction (IMHB^1^ and IMYB^1^) of the different compounds were analyzed, those compounds with two simultaneous and identical interactions either two intramolecular hydrogen bonds (IMHB^2^) or two intramolecular chalcogen bonds (IMYB^2^) were then examined. All the relevant structural data are summarized in Table 3 and the molecular graphs gathered in Figure S2.

As observed, all the compounds with IMHB in which the O acts as a HB donor show a shortening in the intramolecular H···Y distance ranging from –0.002 (OH:Se_14_^np^) to –0.061 Å (OH:S_15_^np^). The opposite is true for the IMHB with S and Se donor, in which the H···O distance increases considerably. Additionally, it is observed that those compounds with shorter distances exhibit more linear interactions, i.e., Y–H···O closer to 180° than those with larger distances, which clearly present narrower angles. In fact, in most of the cases, the Y–H···O is so narrow (97.2° to 110.4°), that it cannot even be considered as a standard HB (>120°). However, the out-of-plane deformation, corresponding to the C–Y···Y**′**–C dihedral angle, is not so dramatic as one should expect. It seems that the SH and SeH groups tend to rotate and drag the H atom out of the molecular plane destabilizing the interaction, rather than opening the dihedral angle in order to accommodate the H atom between the S(Se) and the O acceptor, as happened in the compounds with a single interaction.

In the case of chalcogen bonded compounds (IMYB^2^), there is a reduction in the intramolecular Y···O distances (up to 0.049 Å) which may indicate an increase in the strength of the interaction. No significant variations are observed in the H–Y···O angles with respect to the compounds with a single interaction. Further, no out-of-plane deformation is observed in the IMYB^2^ compounds, since all the interacting atoms are kept within the molecular plane defined by the naphthalene backbone.

The relative energies of each family are reported in Table 4, including the interaction energy, E_int_ and E_iso_ obtained through the partition of Scheme 2 and the deformation energy E_def_. As observed in Table 4, the OH:O compound with the HB donors in the different rings (OH:O_15_) is 2.0 kJ·mol^−1^ more stable than the one with the HB donors in the same ring (OH:O_14_). In the case of S and Se derivatives, those compounds with two simultaneous interactions in which the O atom acts as HB donor are the most stable, particularly those with the donor in different rings. In fact, the differences between OH:S_14_ and OH:S_15_ are 11.9 kJ·mol^−1^ in both cases, while in the OH:O compound the difference was only 2.0 kJ·mol^−1^. Selenium derivatives show similar variations to sulfur compounds. The relative stability order observed is OH:Y < O:Y < O:HY, where chalcogen bonded systems are more stable than IMHB systems with the chalcogen atom (S or Se) acting as HB donor. If the E_int_ are analyzed, negative values of the interaction energies are only observed in OH:Y compounds, while the reverse is seen for the rest of the compounds. The E_int_ are in all cases, more negative in the 1,5 compounds than in the 1,4 ones, indicating a destabilization on the interaction when both donors are located in the same ring. The two only exceptions correspond to O:HS_14_ and O:HS_15_ where the latter is more stable than the former.

How is the interaction energy of the IMHB^2^ and IMYB^2^ systems compared with the respective IMHB^1^ and IMYB^1^ systems? In compounds with oxygen as HB donor, OH:Y_14_ systems show smaller interaction energy than their corresponding OH:Y indicating anti-cooperativity. However, in those with the HB donor (O) in different rings, OH:Y_15_, E_int_ is more negative than in OH:Y. Furthermore, in OH:S_15_ and OH:Se_15_ the interaction energies found in sulfur derivatives, −19.4 and −20.7 kJ·mol^−1^ (OH:S_15_^p^ and OH:S_15_^np^ respectively) and selenium derivatives, −25.5 and −26.7 kJ·mol^−1^ (OH:Se_15_^p^ and OH:S_15_^np^ respectively) are more than twice as much as their corresponding OH:S and OH:Se. This is evidence of the IMHB cooperativity in these particular systems. However, these features were only found in these compounds while the rest show more positive E_int_.

As occurred in the IMHB^1^ and IMYB^1^ compounds, MP2/CBS interation energies are very close to those obtained at the MP2/aug-cc-pVTZ computational level, which again, indicates the convergency of the basis set (Table S2).

### 2.3. Atoms in Molecules (AIM) and Natural Bond Orbital (NBO) Analysis

**Figure 2:**
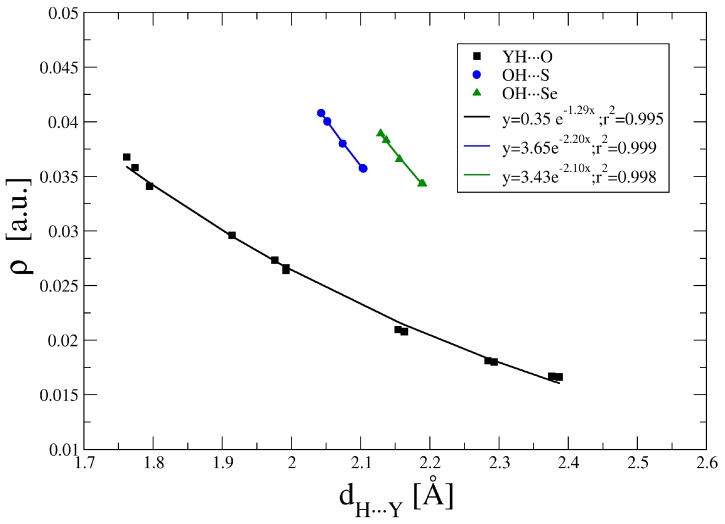
Electron density at the hydrogen bonds (HB) critical points vs. the interatomic distance.

The topological analysis of the electron density within the AIM method shows the presence of BCPs between the interacting groups both in the HB and YB dispositions (Figure S2). The values of the electron density at the BCPs are gathered in Table S3. For the hydrogen bonded complexes, the ρ_BCP_ ranges between 0.041 and 0.017 a.u. Exponential relationships between the ρ_BCP_ and the interatomic distance (Figure 2) are found in agreement with previous reports for hydrogen bonds [55,56,57,58,59] or other weak interactions [6,60]. The correlations show larger values of ρ_BCP_ as the size of the HB acceptor atom becomes larger (Se > S > O). In all the HB cases studied here, ∇^2^ρ_BCP_ is positive (between 0.15 and 0.06 a.u.) and *H*_BCP_ is positive for all the O···H contacts while negative for the S/Se···H as an indication that in these cases they have a partial covalent character [61].

The small number of chalcogen-chalcogen BCPs (three O:S and three O:Se) does not allow correlations to be attempted with another parameter. The values of ρ_BCP_ are between 0.022 and 0.019 au. with ∇^2^ρ_BCP_ and *H*_BCP_ positive in all cases. As in the case of the HBs, the ρ_BCP_ values of the O:Se are larger than those of the O:S for analogous interatomic distances.

**Figure 3:**
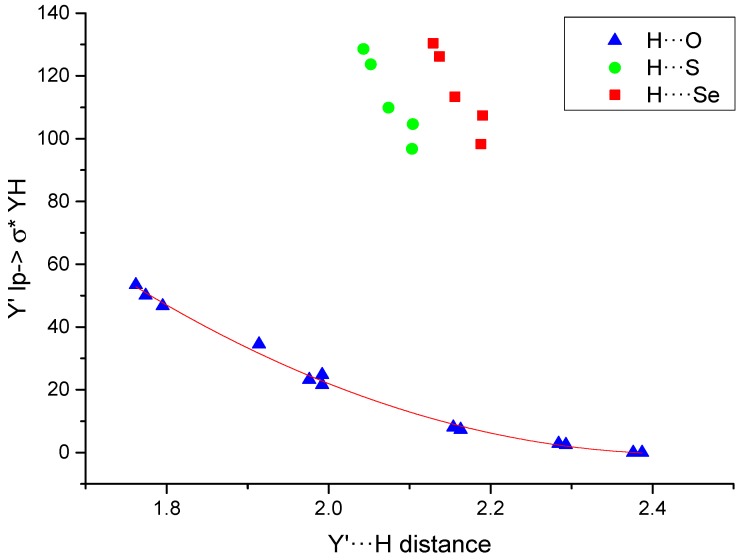
Y’_lp_→σ*HY (kJ·mol^−1^) vs. the Y’···H distance (Å). The fitted second order polynomial curve for the systems with H···O interaction is shown.

The NBO analysis shows stabilization due to the interactions between the double occupied lone pair of the electron donor group and the empty orbital of the electron acceptor (Table 5). The stabilization in the systems with an O···H interaction can reach 53 kJ·mol^−1^, while those with S/Se···H contacts show values between 96 and 130 kJ·mol^−1^. The representation of these values vs. the interatomic distances (Figure 3) shows an excellent second order polynomial relationship for the H···O values (R^2^ = 0.996). Similar relationships have already been found in another type of interaction [62,63]. In addition, it can be observed that the values obtained for the H···S and H···Se increase with the size of the HB acceptor as in the case of the electron density.

The stabilization obtained for the YB contacts are smaller than those obtained for the HBs lying between 11–13 and 15–18 kJ·mol^−1^ for the O:S and O:Se interactions, respectively. It is also worth mentioning that in O:O systems (both IMHB^1^ and IMHB^2^) donations from O_lp_ into the σ*OH antibonding orbitals were found.

**Figure 4:**
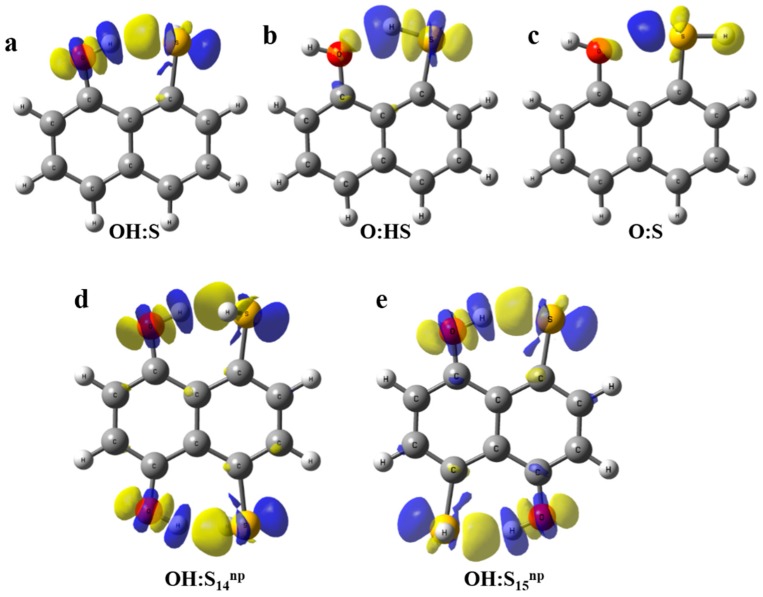
Electron Density Shifts maps at 0.001 a.u. calculated for complexes (**a**) OH:S, (**b**) O:HS, (**c**) O:S, (**d**) OH:S_14_^np^ and (**e**) OH:S_15_^np^.

In order to provide a visualization of the changes in the electron density upon interaction, electron density shift maps (EDS) were obtained using the fragmentation Scheme 2, Equation (3), and are plotted in Figure 4. Blue areas correspond to negative values of the electron density, i.e., areas with a decrease on the electron density. On the other hand, positive (yellow) regions indicate areas with an increment of the electron density when the interaction occurs. Figure 4a–c shows OH:O, O:HS, and O:S systems. As observed, the OH:O compound presents a positive (yellow) region between the H and O atoms, which is consistent with the hydrogen bond interaction. Additionally, blue areas around hydrogen indicate a depletion of the electron density towards the intramolecular region. In the O:HS system, the positive area between O and H is very small and consistent with the relative strength of the interaction. The same occurs in O:S in which the positive area nearby the O atom is even smaller. When the systems with two simultaneous interactions are taken into account (Figure 4d,e) similar electron density patterns are observed to the OH:O system. Despite the interaction found in the former being stronger than in the latter, the EDS maps do not reflect apparent differences, which may lead to evaluation of the relative strength between both systems.

**Figure 5:**
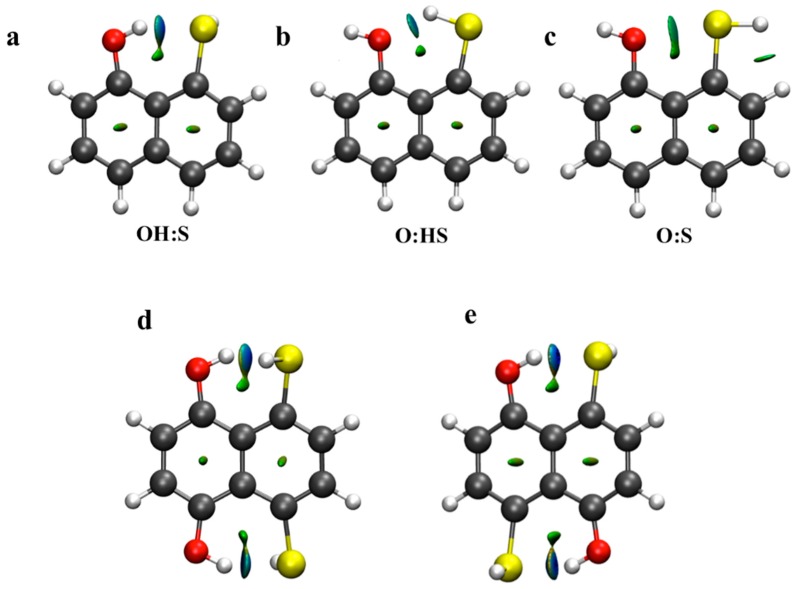
NCI plots of non-covalent interaction for complexes (**a**) **OH:S**, (**b**) **O:HS**, (**c**) **O:S**, (**d**) **OH:S**_14_^np^ and (**e**) **OH:S**_15_^np^. Blue and green areas correspond to λ_2_ > 0 (strongly attractive) and λ_2_ ≈ 0 (weak) respectively. λ_2_ is one of the three eigenvalues of the electron density Hessian with λ_1_ ≤ λ_2_ ≤ λ_3_.

Additionally, non-covalent index plots were also evaluated for the same systems. In Figure 5, blue regions are representative of strong and attractive interactions while green areas indicate weak attractive interactions. As observed, a small blue-green (λ_2_ ≈ 0) area is located between H and S atoms in the OH:S systems, while smaller and green areas are also found in the O:HS and O:S systems. This indicates the relative strength of the non-covalent interactions. Further, those areas become stronger and are characterized by blue color (λ_2_ >0) in the OH:S_14_^np^ and OH:S_15_^np^ systems. However, and as occurred with the EDS maps, the relative strength between both systems with two simultaneous HBs is not appreciable.

A search on the CSD database shows the presence of 38 crystals (46 unique structures) with 1,8-dihydroxynaphthalene structure (Table S4). The analysis of the H–O–C–C1a dihedral angles shows that 29 structures are consistent with the presence of a HB (the absolute value of one dihedral angle smaller than 30° and the other larger than 150°) while no structure is consistent with a chalcogen bond (both dihedral angles larger than 150°). The H···O distances in those structures with a HB range between 1.58 and 1.97 Å, the average being 1.83 Å and the average OH···O angle 145.6°, which are very close to the ones listed in Table 1 and Table 3. For the rest of the systems considered in this article, no structures were found in the CSD.

## 3. Materials and Methods

The structures of the systems were optimized at the MP2 [64]/aug-cc-pVDZ [65,66]. Harmonic vibrational frequencies were computed at the same level used for the geometry optimizations in order to classify the stationary points either as local minima or transition states (TS). Calculations were performed using the Gaussian09 program [67]. The interaction energy between the interacting atoms was obtained through the isodesmic reaction shown in Scheme 2 in two different ways: (a) keeping the resulting fragment fixed in the optimized geometry of the HY:Y′H system, in which the group YH and Y′H were substituted by a hydrogen atom located in the same bond axis as the O, S and Se atom with a C–H distance of 1.1 Å (E_b_), and (b) optimizing the geometry of the isolated fragments, (E_iso_). The differences between both quantities correspond to the deformation energy, in other words, to the penalty or re-organization energy. 

In order to provide more accurate energies, the interaction energies were also estimated at the MP2/CBS (complete basis set) limit using the method of Helgaker et al. [68,69] from the calculated energies with the aug-cc-pVDZ and aug-cc-pVTZ basis sets:

(1) $$E_{X}^{HF}{\;=\;E}_{\mathrm{CBS}}^{\mathrm{HF}}+A\;e^{-\propto X}$$

(2) $$E_{X}^{MP2\;}{=\;E}_{\mathrm{CBS}}^{\mathrm{MP}2}+B\;X^{-3}$$

where *E*_X_ and *E*_CBS_ are the energies for the aug-cc-pVDZ and aug-cc-pVTZ basis set (X = 2 and 3, respectively) and for the complete basis set, respectively.

The Atoms in Molecules (AIM) methodology [70,71] was used to analyze the electron density of the systems with the AIMAll program [72]. The Natural Bond Orbital (NBO) method [73] was employed to evaluate atomic charges using the NBO-6 program, and to analyze charge–transfer interactions between occupied and unoccupied orbitals. 

The NCI (non-covalent interactions) index, based on the reduced gradient of the electron density, was calculated to identify attractive and repulsive interactions with the NCI program [74] and was plotted with the VMD program [75]. 

The intramolecular electron density shift (EDS) was obtained using the fragmentation scheme reported in ref. [76]. This method proposes the calculation of the EDS of the intramolecular interaction by comparing the electron density of the interacting moieties substituted by hydrogen atoms as shown in Scheme 2. The EDS is calculated using Equation (3)

EDS = ρ(YH:Y′H) − ρ(YH) − ρ(Y′H) + ρ(HH)
(3)

The CSD database (version 5.38) [77] was explored in order to find experimental structures similar to those considered here.

## 4. Conclusions 

The competition between intramolecular hydrogen and chalcogen bonds was studied by means of MP2/aug-cc-pVTZ calculations in system with (a) one interaction, and (b) two identical interactions acting simultaneously.

Systems with one interaction (IMHB^1^ and IMYB^1^), show negative interaction energies only in those systems with the O–H group acting as a hydrogen donor, while the chalcogen interaction and hydrogen bonds with Y–H (Y = S, Se) group as HB donor present positive interaction energies revealing unfavorable interactions. The relative energies between the different families with the same atoms indicate that the relative stability is as follows: OH:Y > O:Y > O:HY, OH:Y being the most stable compound.

In the case of systems with two simultaneous interactions (IMHB^2^ and IMYB^2^), negative values of the E_int_ were only found for OH:Y compounds as occurred in IMHB^1^. When the HB donors are located in the same ring (OH:Y_14_), the interaction energies found are less than in the parent compound, while if the HB donors are located in different rings (OH:Y_15_) the interaction energies found are greater than in the IMHB^1^ systems. Furthermore, in OH:S_15_ and OH:Se_15_ compounds, the interaction energies found are more than twice those of OH:S and OH:Se respectively, indicating cooperative effects.

Atoms in molecules analysis of ρ_BCP_, ∇^2^ρ_BCP_ and *H*_BCP_ revealed that HBs present partial covalent character. Exponential correlation between electron density at the BCP and intramolecular distances was also found. Additionally, NBO results indicate large second orbital interaction values from the lone pair belonging to the electron donor to the antibonding orbital H–Y’. These are particularly large in HBs with oxygen acting as electron donor. Further, NBO data are in agreement with the energetics found for the systems studied.

Electron density shift maps and non-covalent index plots were used to assess a visual description of the intramolecular interactions. Despite the fact that the electron density patterns found indicate a preference for the HBs rather than for the YBs, it is not possible to discriminate between systems with HB donors located in different rings. The same occurs with NCI plots.

## Figures and Tables

**Table 1:** Bond lengths (Å), Y–H···Y′ angles and dihedral C–Y···Y′–C angles (°) for the different compounds at MP2/aug-cc-pVDZ computational level.

IMHB^1^	H···Y′	Y–H···Y′	C–Y···Y′–C
OH:O	1.795	144.2	0.0
OH:S	2.104	152.8	0.0
OH:Se	2.190	154.6	0.0
O:HS	1.914	134.6	0.0
O:HSe ^a^	1.992	128.0	0.0
IMYB^1^	O···Y	O···Y′–H	C–O···Y′–C
O:O	2.574	163.4	0.0
O:S	2.702	170.2	0.0
O:Se	2.734	165.2	0.0

^a^ O:HSe presents an imaginary frequency (118 cm^−1^), which corresponds to a rotation around the C–Se axis. This is a transition state which connects with OH:Se minimum. However, for the sake of comparison this structure will be considered.

**Table 2:** Relative energies, E_rel_, and interaction energies, E_iso_, E_int_, and deformation energies E_def_, (kJ·mol^−1^) for the different compounds at MP2/CBS computational level.

Compound	E_int_	E_iso_	E_def_	E_rel OO_	E_rel SO_	E_rel SeO_
IMHB^1^						
OH:O	–12.6	–3.6	9.0	0.0		
OH:S	–10.0	4.1	14.1		0.0	
OH:Se	–12.3	3.7	16.0			0.0
O:HS	4.1	18.9	14.9		14.9	
O:HSe	6.1	25.5	19.5			21.9
IMYB^1^						
O:O	14.6	23.7	9.2	27.8		
O:S	9.1	14.6	5.4		10.5	
O:Se	5.2	12.5	7.2			8.8

**Table 3:** Bond lengths (Å), Y–H···Y’ angles and dihedral Z–C–C–Z′ angles (°) for the different compounds at MP2/aug-cc-pVDZ computational level.

	H···Y′	ΔH···Y′ ^b^	Y–H···Y′	C–Y···Y′–C
IMHB^2 a^				
OH:O_14_	1.774	–0.021	143.8	0.0
OH:O_15_	1.762	–0.033	144.5	0.0
OH:S_14_^p^	2.074	–0.030	151.2	5.9
OH:S_14_^np^	2.103	–0.001	148.0	12.0
OH:S_15_^p^	2.052	–0.053	152.9	7.9
OH:S_15_^np^	2.043	–0.061	153.7	5.0
OH:Se_14_^p^	2.156	–0.033	152.9	5.7
OH:Se_14_^np^	2.188	–0.002	149.0	12.1
OH:Se_15_^p^	2.137	–0.053	154.5	8.0
OH:Se_15_^np^	2.129	–0.061	155.3	5.0
O:HS_14_^p^	1.992	0.078	122.6	14.1
O:HS_14_^np^	1.976	0.062	123.7	11.9
O:HS_15_^p^	2.163	0.249	110.0	13.6
O:HS_15_^np^	2.154	0.240	110.4	15.1
O:HSe_14_^p^	2.284	0.292	102.3	13.4
O:HSe_14_^np^	2.293	0.300	101.6	11.7
O:HSe_15_^p^	2.387	0.395	97.2	16.0
O:HSe_15_^np^	2.376	0.383	97.7	15.6
IMYB^2^ ^a^				
HO···SH	O···Y'	ΔO··Y′ ^b^	O···Y′–H	C–O···Y′–C
O:O	2.525	–0.049	163.2	0.0
O:S_14_	2.654	–0.049	169.6	0.0
O:S_15_	2.657	–0.046	169.7	0.0
O:Se_14_	2.690	–0.044	164.8	0.0
O:Se_15_	2.694	–0.040	164.9	0.0

^a^ Subscripts _14_ and _15_ indicate the position of the OH group. Superscripts ^p^ and ^np^ indicates whether the Hs bond to S(Se) atom are pointing towards the same side (^p^) or towards different sides (^np^) of the molecular plane. ^b^ ΔH···Y**′** and ΔO···Y**′** distances are obtained with respect to the single interacting compounds.

**Table 4:** Relative, E_rel_, and interaction energies, E_iso_, E_b_, and deformation energies E_def_, (kJ·mol^−1^) for the different compounds at MP2/CBS computational level.

IMHB^2^	E_int_	E_iso_	E_def_	E_rel SO_	E_rel SeO_
OH:O_14_	–11.8	9.1	20.9	4.3 ^a^	
OH:O_15_	–13.8	4.8	18.5	0.0 ^a^	
OH:S_14_^p^	–7.5	27.3	34.9	19.4	
OH:S_14_^np^	–8.8	24.5	33.4	16.6	
OH:S_15_^p^	–19.4	7.1	26.5	0.0	
OH:S_15_^np^	–20.7	7.5	28.2	0.4	
OH:Se_14_^p^	–12.0	26.1	38.2		20.1
OH:Se_14_^np^	–10.3	23.8	34.1		17.8
OH:Se_15_^p^	–25.5	5.2	30.7		0.0
OH:Se_15_^np^	–26.7	5.9	32.6		0.7
O:HS_14_^p^	19.4	54.5	35.1	46.6	
O:HS_14_^np^	20.1	55.0	34.8	47.0	
O:HS_15_^p^	24.9	53.1	28.2	46.1	
O:HS_15_^np^	23.8	52.1	28.3	45.1	
O:HSe_14_^p^	30.8	62.5	31.6		56.4
O:HSe_14_^np^	32.2	62.9	30.6		56.8
O:HSe_15_^p^	27.4	55.9	28.5		50.7
O:HSe_15_^np^	27.5	55.6	28.0		50.4
IMYB^2^					
HO···SH					
O:O	52.9	65.9	13.0	61.2 ^a^	
O:S_14_	27.5	40.0	12.5	32.1	
O:S_15_	27.4	38.1	10.8	31.0	
O:Se_14_	16.8	32.3	15.5		26.2
O:Se_15_	17.3	31.0	13.7		25.9

^a^ Relative energy only between OH:O_14_ and OH:O_15_ compounds. Superscripts ^p^ and ^np^ indicates whether the Hs bond to S(Se) atom are pointing towards the same side (^p^) or towards different sides (^np^) of the molecular plane.

**Table 5:** Natural Bond Orbital (NBO) stabilization (kJ·mol^−1^) due to the donation from the chalcogen lone pair (Y′_lp_) to the antibonding orbital (Y′_lp_→σ*HY) in the hydrogen bonds (HB) systems and O_lp_→σ*YH in the chalcogen bond (YB) systems.

HB		YB	
	Y′_lp_→σ*HY		O_lp_→σ*YH
OH:O	46.69	O:O	-
OH:S	104.63	O:S	10.58
OH:Se	107.38	O:Se	15.26
O:HS	34.53		
O:HSe	24.79		
	Y′_lp_→σ*HY		O_lp_→σ*YH
OH:O_14_	50.03	O:O	-
OH:O_15_	53.42	O:S_14_	12.71
OH:S_14_^p^	109.85	O:S_15_	12.46
OH:S_14_^np^	96.73	O:Se_14_	17.68
OH:S_15_^p^	123.69	O:Se_15_	17.35
OH:S_15_^np^	128.58		
OH:Se_14_^p^	113.36		
OH:Se_14_^np^	98.27		
OH:Se_15_^p^	126.19		
OH:Se_15_^np^	130.37		
O:HS_14_^p^	21.57		
O:HS_14_^np^	23.24		
O:HS_15_^p^	7.32		
O:HS_15_^np^	8.03		
O:HSe_14_^p^	2.84		
O:HSe_14_^np^	2.47		
O:HSe_15_^p^	0.00		
O:HSe_15_^np^	0.00		

Superscripts ^p^ and ^np^ indicates whether the Hs bond to S(Se) atom are pointing towards the same side (^p^) or towards different sides (^np^) of the molecular plane.

## Supplementary Materials

The following are available online at: http://www.mdpi.com/1420-3049/22/2/227/s1, Table S1. Relative energies, E_rel_, and interaction energies, E_iso_, E_int_, and deformation energies E_def_, (kJ·mol^−1^) for the different IMHB^1^ and IMYB^1^ compounds at MP2/aug-cc-pVTZ computational level. Table S2. Relative, E_rel_, and interaction energies, E_iso_, E_b_, and deformation energies E_def_, (kJ·mol^−1^) for the different IMHB^2^ and IMYB^2^ compounds at MP2/aug-cc-pVTZ computational level. Table S3: Intramolecular distance, in Å, electron density, Laplacian, G, V, and total energy density, H at the bond critical point, in a.u. at the MP2/aug-cc-pVDZ computational level. Table S4: Refcode and geometrical parameters (Å and °) of CSD structures consistent with the presence of a OH···O HB interaction in 1,8-dihydroxynapthalene. Figure S1. Molecular electrostatic potential on the 0.001 a.u. electron density isosurface for at the MP2/aug-cc-pVDZ computational level. Figure S2. Molecular graphs corresponding to those systems with two simultaneous intramolecular interactions at the MP2/aug-cc-pVDZ computational level.
