# Supplementary material for: Theoretical Study of Intramolecular Interactions in Peri-Substituted Naphthalenes: Chalcogen and Hydrogen Bonds
Source: Molecules. 2017 Feb 2;22(2):227. doi: 10.3390/molecules22020227 (PMC6155622; doi:10.3390/molecules22020227)
Supplement: Supplementary file 1 [file molecules-22-00227-s001.pdf]

Supplementary Materials: Theoretical Study of Intramolecular Interactions in Peri-Substituted Naphthalenes: Chalcogen and Hydrogen Bonds

Goar Sánchez–Sanz, Ibon Alkorta and José Elguero

Table S1. Relative energies, E_{rel}, and interaction energies, E_{iso}, E_{int}, and deformation energies E_{def}, (kJ·mol^{-1}) for the different IMHB[1] and IMYB[1] compounds at MP2/aug-cc-pVTZ computational level.

Compound	E_{int}	E_{iso}	E_{def}	$E_{rel\ OO}$	$E_{rel\ SO}$	$E_{rel\ SeO}$
IMHB[1]						
OH:O	–12.3	–3.5	8.8	0.0		
OH:S	–8.9	4.1	13.1		0.0	
OH:Se	–10.4	4.4	14.7			0.0
O:HS	4.1	19.2	15.1		16.0	
O:HSe	6.6	25.7	19.1			20.7
IMYB[1]						
O:O	17.8	23.3	5.5	26.8		
O:S	9.3	14.6	5.3		11.1	
O:Se	5.4	12.1	6.7			5.5

Table S2. Relative, E_{rel}, and interaction energies, E_{iso}, E_b, and deformation energies E_{def}, (kJ·mol^{-1}) for the different IMHB[2] and IMYB[2] compounds at MP2/aug-cc-pVTZ computational level.

IMHB[2]	E_{int}	E_{iso}	E_{def}	$E_{rel\ SO}$	$E_{rel\ SeO}$
OH:O_{14}	–10.6	9.0	19.7	3.8[a]	
OH:O_{15}	–14.1	5.2	19.3	0.0[a]	
OH:S_{14}^{p}	–4.7	27.8	32.5	19.2	
OH:S_{14}^{np}	–5.0	25.3	30.3	16.7	
OH:S_{15}^{p}	–18.4	8.0	26.4	0.0	
OH:S_{15}^{np}	–18.8	8.8	27.6	0.8	
OH:Se_{14}^{p}	–7.4	28.2	35.6		19.5
OH:Se_{14}^{np}	–6.5	25.6	32.2		16.9
OH:Se_{15}^{p}	–21.3	8.2	29.6		0.0
OH:Se_{15}^{np}	–21.8	9.2	31.0		1.0
O:HS_{14}^{p}	21.7	55.3	33.6	46.7	
O:HS_{14}^{np}	22.6	55.8	33.3	47.2	
O:HS_{15}^{p}	25.7	52.7	26.9	44.7	
O:HS_{15}^{np}	24.0	51.8	27.8	43.8	
O:HSe_{14}^{p}	32.5	62.2	29.7		53.5
O:HSe_{14}^{np}	34.1	62.7	28.6		54.0
O:HSe_{15}^{p}	28.6	55.7	27.1		47.5
O:HSe_{15}^{np}	28.5	55.2	26.7		47.0
IMYB[2]					
HO···SH					
O:O	52.9	65.1	12.2	59.9[a]	
O:S_{14}	28.9	41.2	12.3	32.6	
O:S_{15}	28.2	39.6	11.4	31.6	
O:Se_{14}	18.7	33.2	14.5		24.5
O:Se_{15}	17.7	31.5	13.8		23.3

[a] Relative energy only between OH:O_{14} and OH:O_{15} compounds.

Table S3. Intramolecular distance, in Å, electron density, Laplacian, G, V, and total energy density, H at the bond critical point, in a.u. at the MP2/aug-cc-pVDZ computational level.

**IMHB^1**	**H···Y'**	**ρ**	**$\nabla^2\rho$**	**G**	**V**	**H**
OH:O	1.795	0.0341	0.1356	0.0321	−0.0303	0.0018
OH:S	2.104	0.0357	0.0774	0.0219	−0.0244	−0.0025
OH:Se	2.190	0.0343	0.0635	0.0190	−0.0222	−0.0032
O:HS	1.914	0.0296	0.1026	0.0248	−0.0240	0.0008
O:HSe	1.992	0.0264	0.0866	0.0213	−0.0209	0.0004
**IMYB^1**	**O···Y**	**ρ**	**$\nabla^2\rho$**	**G**	**V**	**H**
O:O	2.574	0.0160	0.0718	0.0162	−0.0144	0.0018
O:S	2.702	0.0194	0.0746	0.0170	−0.0154	0.0016
O:Se	2.734	0.0204	0.0733	0.0173	−0.0162	0.0011
**IMHB^2**	**H···Y′**	**ρ**	**$\nabla^2\rho$**	**G**	**V**	**H**
OH:O_{14}	1.774	0.0358	0.1438	0.0342	−0.0324	0.0018
OH:O_{15}	1.762	0.0368	0.1477	0.0352	−0.0335	0.0017
OH:S_{14}^{p}	2.074	0.0380	0.0818	0.0237	−0.0270	−0.0033
OH:S_{14}^{np}	2.103	0.0358	0.0786	0.0224	−0.0251	−0.0027
OH:S_{15}^{p}	2.052	0.0400	0.0827	0.0247	−0.0287	−0.0040
OH:S_{15}^{np}	2.043	0.0408	0.0835	0.0252	−0.0295	−0.0043
OH:Se_{14}^{p}	2.156	0.0366	0.0671	0.0207	−0.0247	−0.0040
OH:Se_{14}^{np}	2.188	0.0343	0.0648	0.0196	−0.0229	−0.0034
OH:Se_{15}^{p}	2.137	0.0383	0.0671	0.0214	−0.0260	−0.0046
OH:Se_{15}^{np}	2.129	0.0389	0.0676	0.0217	−0.0265	−0.0048
O:HS_{14}^{p}	1.992	0.0266	0.0918	0.0224	−0.0219	0.0006
O:HS_{14}^{np}	1.976	0.0273	0.0946	0.0231	−0.0225	0.0006
O:HS_{15}^{p}	2.163	0.0208	0.0747	0.0177	−0.0168	0.0010
O:HS_{15}^{np}	2.154	0.0210	0.0749	0.0178	−0.0169	0.0009
O:HSe_{14}^{p}	2.284	0.0181	0.0660	0.0151	−0.0138	0.0014
O:HSe_{14}^{np}	2.293	0.0180	0.0661	0.0151	−0.0137	0.0014
O:HSe_{15}^{p}	2.387	0.0166	0.0626	0.0139	−0.0122	0.0017
O:HSe_{15}^{np}	2.376	0.0167	0.0632	0.0140	−0.0123	0.0018
**IMYB^2**	**O···Y**	**ρ**	**$\nabla^2\rho$**	**G**	**V**	**H**
O:O	2.525	0.0177	0.0818	0.0183	−0.0161	0.0022
O:S_{14}	2.654	0.0214	0.0829	0.0190	−0.0174	0.0017
O:S_{15}	2.657	0.0213	0.0824	0.0189	−0.0172	0.0017
O:Se_{14}	2.690	0.0223	0.0802	0.0191	−0.0181	0.0010
O:Se_{15}	2.694	0.0221	0.0795	0.0189	−0.0179	0.0010

Table S4. Refcode and geometrical parameters (Å and °) of CSD structures consistent with the presence of a OH···O HB interaction in 1,8-dihydroxynapthalene.

Refcode	H···O Distance	O-H···O Angle	$H^1O^1C^1C^{8a}$ Dihedral	$H^8O^8C^8C^{8a}$ Dihedral
BABXUA	1.900	142.5	−3.8	177.6
BODWAS	1.912	147.4	−3.6	−177.1
FAXWIL	1.853	145.3	−1.5	−178.2
FOKVIL	1.890	145.8	−2.4	−174.7
GACPIM	1.862	146.2	6.3	−175.1
GACPIM	1.849	146.8	−3.8	−178.8
GERMIC	1.803	136.5	7.6	−169.8
GERMIC	1.965	148.6	−6.8	171.6
HEKWAV	1.767	146.5	3.0	−179.3
HEKWAV01	1.744	146.8	5.3	−177.3
HUNGIN	1.898	144.6	−15.4	177.4
HURWAU	1.782	145.2	−1.9	177.7
IZOXAX	1.969	145.2	0.6	−170.7
JAYLUR	1.749	147.1	1.3	−173.0
MAPHES	1.758	148.2	0.3	−153.5
MEYREP	1.857	146.1	1.3	172.9
MOSTAP	1.879	135.5	10.3	172.8
MRUBFN01	1.875	145.6	2.9	−179.4
OWELOS	1.609	154.2	−0.5	−166.2
PILLMA	1.811	150.0	13.9	−164.1
RUBFUS02	1.885	144.9	8.2	177.5
SAGSUR	1.951	144.1	3.2	−175.6
SOKSER	1.804	144.4	8.1	−167.4
TUXBOD	1.875	145.8	−1.0	176.7
VISONA	1.580	147.4	−9.9	174.7
VOBLUV	1.887	144.1	−4.3	178.2
VOBLUV	1.839	142.6	−5.8	177.3
VOBMEG	1.809	148.9	−1.5	−179.8
VOBMEG	1.823	145.0	−6.1	−179.0
Max.	1.969	154.2		
Min.	1.580	135.5		
Aver.	1.834	145.6		

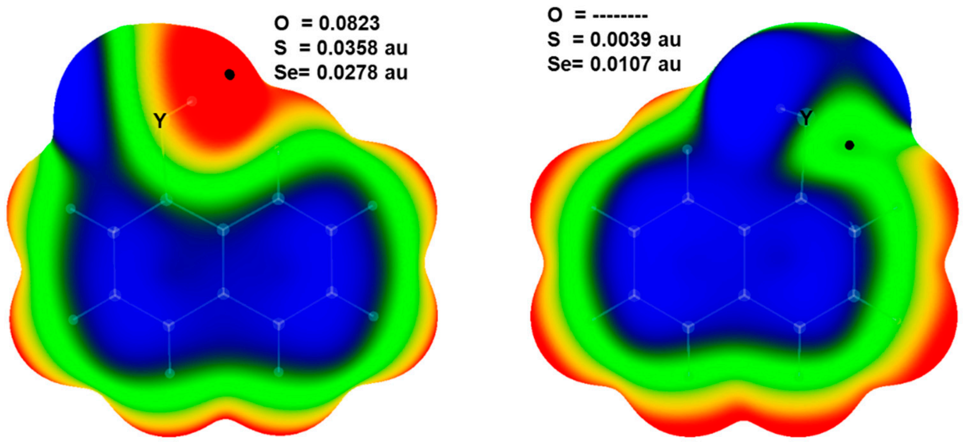

Figure S1. Molecular electrostatic potential on the 0.001 a.u electron density isosurface for at the MP2/aug-cc-pVDZ computational level.

Compd.	Mol. Graph	Coordinates			
OH:O14	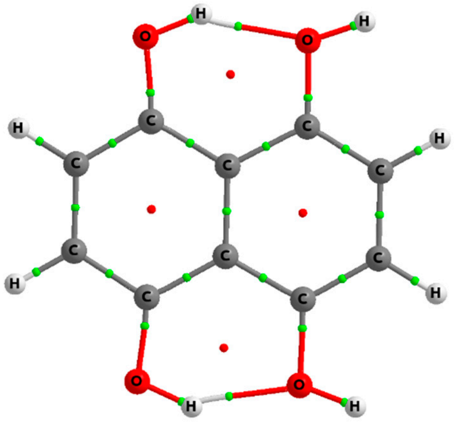	C	−1.5174	−1.1012	0.0000
		C	−0.9525	−2.3673	0.0000
		C	0.4583	−2.5132	0.0000
		C	1.2702	−1.3893	0.0000
		C	1.5540	1.1166	0.0000
		C	0.9585	2.3778	0.0000
		C	−0.4421	2.5223	0.0000
		C	−1.2834	1.4097	0.0000
		C	−0.7204	0.0908	0.0000
		C	0.7332	−0.0595	0.0000
		H	−1.5948	−3.2542	0.0000
		H	0.9055	−3.5128	0.0000
		H	1.6125	3.2537	0.0000
		H	−0.9031	3.5135	0.0000
		O	2.9293	1.0958	0.0000
		H	3.2237	0.1696	0.0000
		O	2.6591	−1.5119	0.0000
		H	2.8924	−2.4511	0.0000
		O	−2.6337	1.6712	0.0000
		H	−3.1119	0.8251	0.0000
		O	−2.9021	−0.9371	0.0000
		H	−3.3228	−1.8087	0.0000
OH:O15	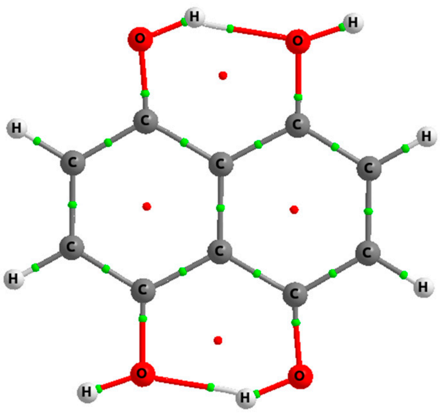	C	0.0100	−1.6838	−0.8876
		C	0.1170	−1.2731	−2.2140
		C	0.2010	0.0994	−2.5398
		C	0.1782	1.0627	−1.5401
		C	0.0438	1.6802	0.9005
		C	−0.0632	1.2697	2.2270
		C	−0.1473	−0.1028	2.5529
		C	−0.1245	−1.0662	1.5533
		C	−0.0164	−0.7106	0.1726
		C	0.0701	0.7069	−0.1595
		H	0.1342	−2.0381	−2.9939
		H	0.2847	0.4067	−3.5882
		H	−0.0803	2.0349	3.0068
		H	−0.2311	−0.4100	3.6013
		O	0.1194	3.0344	0.6898
		H	0.1905	3.1940	−0.2675

		O	0.2605	2.4227	−1.8490
		H	0.3295	2.5219	−2.8091
		O	−0.2070	−2.4262	1.8624
		H	−0.2754	−2.5253	2.8225
		O	-0.0655	-3.0380	-0.6772
		H	-0.1366	-3.1979	0.2800
OH:S14p	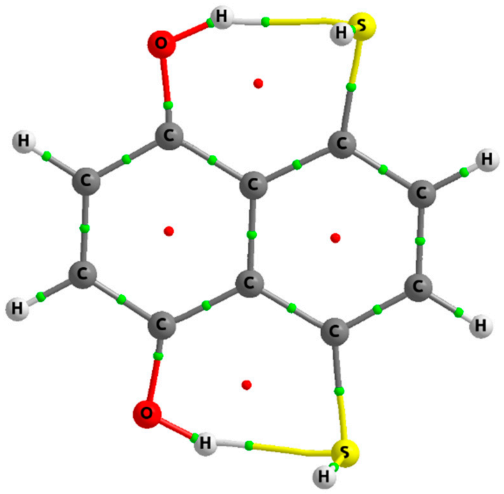	C	−1.4258	−0.0276	−1.4264
		C	−2.6123	−0.1391	−0.7011
		C	−2.6123	−0.1391	0.7011
		C	−1.4258	−0.0276	1.4264
		C	1.1134	0.0819	1.4136
		C	2.3071	0.2109	0.7052
		C	2.3071	0.2109	−0.7052
		C	1.1134	0.0819	−1.4136
		C	−0.1616	0.0253	−0.7402
		C	−0.1616	0.0253	0.7402
		H	−3.5484	−0.1910	−1.2634
		H	−3.5484	−0.1910	1.2634
		H	3.2505	0.2686	1.2543
		H	3.2505	0.2686	−1.2543
		S	1.3409	0.0155	3.2161
		H	1.1398	−1.3192	3.3403
		O	−1.5965	0.0479	2.7872
		H	−0.7272	0.1745	3.2166
		O	−1.5965	0.0479	−2.7872
		H	−0.7272	0.1745	−3.2166
		S	1.3409	0.0155	−3.2161
		H	1.1398	−1.3192	−3.3403
OH:S14np	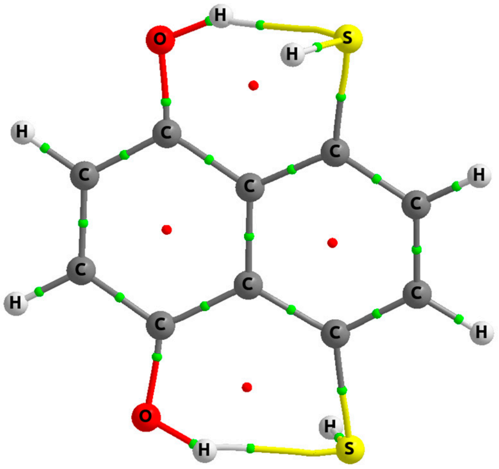	C	0.1510	−1.4180	1.3916
		C	0.0879	−0.6963	2.5840
		C	−0.0879	0.6963	2.5840
		C	−0.1510	1.4180	1.3916
		C	0.0692	1.4111	−1.1412
		C	0.0603	0.7030	−2.3421
		C	−0.0603	−0.7030	−2.3421
		C	−0.0692	−1.4111	−1.1412
		C	0.0277	−0.7376	0.1304
		C	−0.0277	0.7376	0.1304
		H	0.1761	−1.2519	3.5214
		H	−0.1761	1.2519	3.5214
		H	0.1407	1.2494	−3.2857
		H	−0.1407	−1.2494	−3.2857
		S	0.2040	3.2085	−1.3451
		H	1.4363	3.3285	−0.7936
		O	−0.3616	2.7671	1.5447
		H	−0.4162	3.1881	0.6644
		O	0.3616	−2.7671	1.5447
		H	0.4162	−3.1881	0.6644
		S	−0.2040	−3.2085	−1.3451
		H	−1.4363	−3.3285	−0.7936

$OH{:}S_{15}^{p}$	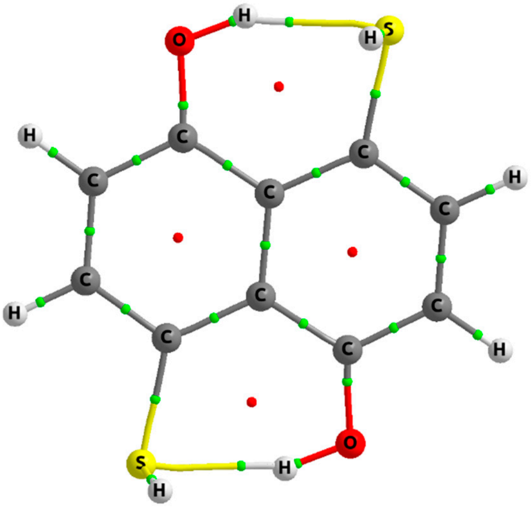	C	1.7826	0.6737	−0.0324
		C	1.5403	2.0487	0.0008
		C	0.2336	2.5542	0.0919
		C	−0.8646	1.6937	0.1071
		C	−1.7826	−0.6737	−0.0324
		C	−1.5403	−2.0487	0.0008
		C	−0.2336	−2.5542	0.0919
		C	0.8646	−1.6937	0.1071
		C	0.6878	−0.2614	0.0296
		C	−0.6878	0.2614	0.0296
		H	2.3891	2.7365	−0.0425
		H	0.0426	3.6287	0.1495
		H	−2.3891	−2.7365	−0.0425
		H	−0.0426	−3.6287	0.1495
		S	−3.5363	−0.2263	−0.1440
		H	−3.5472	0.0662	−1.4681
		O	−2.0709	2.3181	0.2267
		H	−2.7877	1.6472	0.2278
		S	3.5363	0.2263	−0.1440
		H	3.5472	−0.0662	−1.4681
		O	2.0709	−2.3181	0.2267
		H	2.7877	−1.6472	0.2278
$OH{:}S_{15}^{np}$	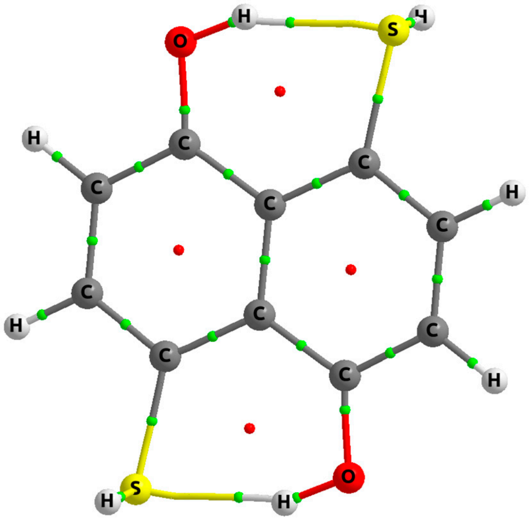	C	1.7853	0.6665	0.0955
		C	1.5439	2.0398	0.1752
		C	0.2379	2.5518	0.1345
		C	−0.8589	1.6992	0.0038
		C	−1.7853	−0.6665	−0.0955
		C	−1.5439	−2.0398	−0.1752
		C	−0.2379	−2.5518	−0.1345
		C	0.8589	−1.6992	−0.0038
		C	0.6870	−0.2643	0.0237
		C	−0.6870	0.2643	−0.0237
		H	2.3950	2.7225	0.2474
		H	0.0481	3.6276	0.1702
		H	−2.3950	−2.7225	−0.2474
		H	−0.0481	−3.6276	−0.1702
		S	−3.5431	−0.2171	−0.1029
		H	−3.6862	−0.0945	1.2400
		O	−2.0581	2.3381	−0.1074
		H	−2.7758	1.6738	−0.1968
		S	3.5431	0.2171	0.1029
		H	3.6862	0.0945	−1.2400
		O	2.0581	−2.3381	0.1074
		H	2.7758	−1.6738	0.1968

OH:Se$_{14}$^{p}

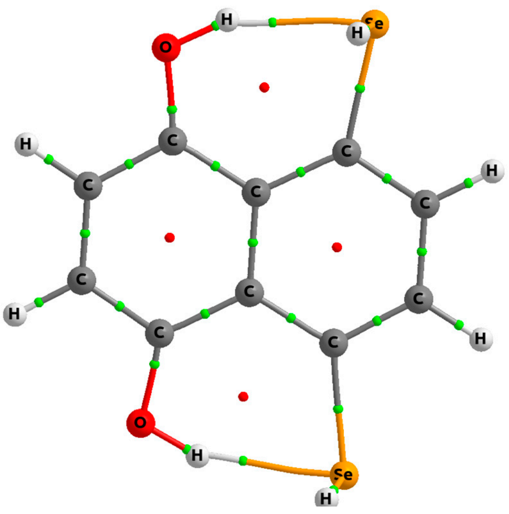

C	−1.4315	−0.0262	−1.4264
C	−2.6163	−0.1488	−0.7003
C	−2.6163	−0.1488	0.7003
C	−1.4315	−0.0262	1.4264
C	1.1086	0.1076	1.4156
C	2.3015	0.2380	0.7050
C	2.3015	0.2380	−0.7050
C	1.1086	0.1076	−1.4156
C	−0.1658	0.0406	−0.7427
C	−0.1658	0.0406	0.7427
H	−3.5514	−0.2114	−1.2631
H	−3.5514	−0.2114	1.2631
H	3.2483	0.2977	1.2481
H	3.2483	0.2977	−1.2481
Se	1.3914	0.0421	3.3454
H	1.1694	−1.4088	3.4564
O	−1.6164	0.0427	2.7855
H	−0.7581	0.1742	3.2349
O	−1.6164	0.0427	−2.7855
H	−0.7581	0.1742	−3.2349
Se	1.3914	0.0421	−3.3454
H	1.1694	−1.4088	−3.4564

OH:Se$_{14}$^{np}

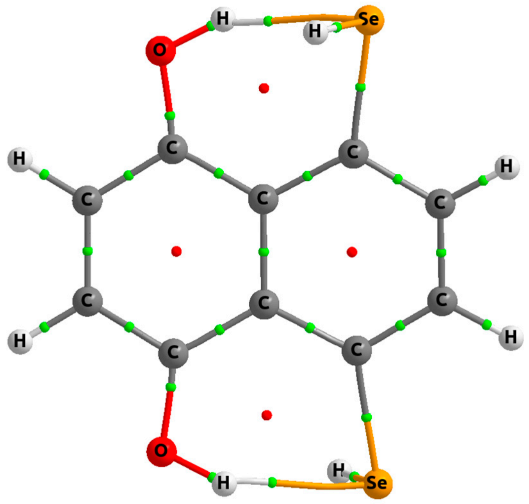

C	0.1635	−1.4163	1.3913
C	0.0954	−0.6945	2.5833
C	−0.0954	0.6945	2.5833
C	−0.1635	1.4163	1.3913
C	0.0619	1.4133	−1.1432
C	0.0593	0.7030	−2.3435
C	−0.0593	−0.7030	−2.3435
C	−0.0619	−1.4133	−1.1432
C	0.0345	−0.7398	0.1281
C	−0.0345	0.7398	0.1281
H	0.1888	−1.2498	3.5204
H	−0.1888	1.2498	3.5204
H	0.1425	1.2432	−3.2904
H	−0.1425	−1.2432	−3.2904
Se	0.2003	3.3382	−1.3982
H	1.5214	3.4589	−0.7607
O	−0.3851	2.7621	1.5566
H	−0.4537	3.1985	0.6851
O	0.3851	−2.7621	1.5566
H	0.4537	−3.1985	0.6851
Se	−0.2003	−3.3382	−1.3982
H	−1.5214	−3.4589	−0.7607

OH:Se$_{15}^{p}$	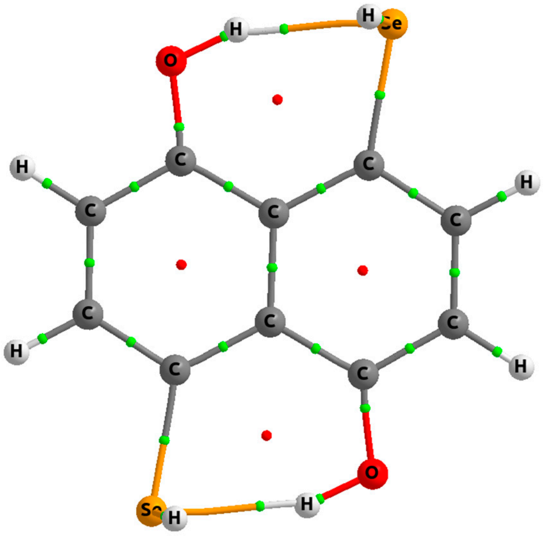	C	1.7824	0.6825	−0.0132
		C	1.5310	2.0571	0.0116
		C	0.2229	2.5552	0.1033
		C	−0.8719	1.6907	0.1205
		C	−1.7824	−0.6825	−0.0132
		C	−1.5310	−2.0571	0.0116
		C	−0.2229	−2.5552	0.1033
		C	0.8719	−1.6907	0.1205
		C	0.6914	−0.2577	0.0465
		C	−0.6914	0.2577	0.0465
		H	2.3722	2.7537	−0.0367
		H	0.0260	3.6290	0.1558
		H	−2.3722	−2.7537	−0.0367
		H	−0.0260	−3.6290	0.1558
		Se	−3.6741	−0.2491	−0.1406
		H	−3.6517	0.0784	−1.5765
		O	−2.0744	2.3221	0.2385
		H	−2.8036	1.6652	0.2372
		Se	3.6741	0.2491	−0.1406
		H	3.6517	−0.0784	−1.5765
		O	2.0744	−2.3221	0.2385
		H	2.8036	−1.6652	0.2372
OH:Se$_{15}^{np}$	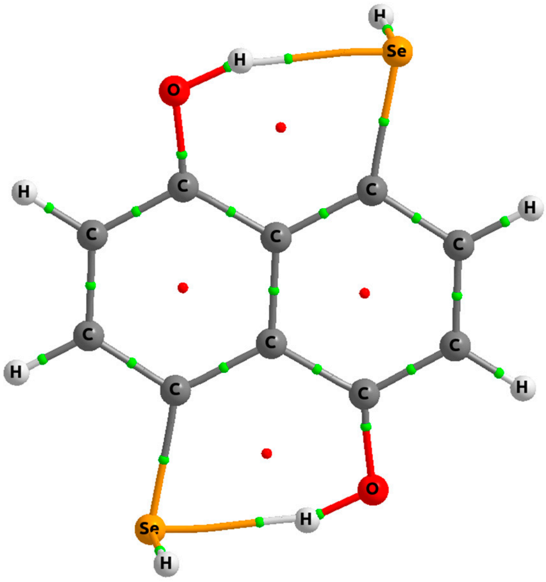	C	1.7863	0.6699	0.1099
		C	1.5401	2.0436	0.1833
		C	0.2342	2.5519	0.1351
		C	−0.8611	1.6985	−0.0011
		C	−1.7863	−0.6699	−0.1099
		C	−1.5401	−2.0436	−0.1833
		C	−0.2342	−2.5519	−0.1351
		C	0.8611	−1.6985	0.0011
		C	0.6897	−0.2627	0.0288
		C	−0.6897	0.2627	−0.0288
		H	2.3855	2.7328	0.2577
		H	0.0418	3.6274	0.1686
		H	−2.3855	−2.7328	−0.2577
		H	−0.0418	−3.6274	−0.1686
		Se	−3.6817	−0.2298	−0.1275
		H	−3.8105	−0.0882	1.3329
		O	−2.0540	2.3480	−0.1162
		H	−2.7860	1.7001	−0.2060
		Se	3.6817	0.2298	0.1275
		H	3.8105	0.0882	−1.3329
		O	2.0540	−2.3480	0.1162
		H	2.7860	−1.7001	0.2060

$O{:}HS_{14}^{p}$

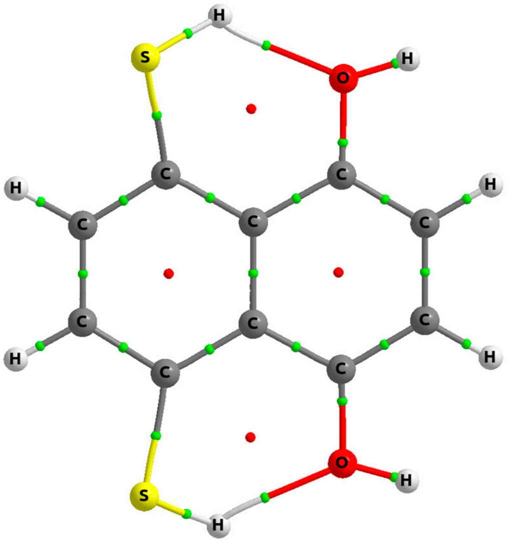

C	0.0320	1.4907	1.4081
C	−0.0238	2.6877	0.7041
C	−0.0238	2.6877	−0.7041
C	0.0320	1.4907	−1.4081
C	0.0249	−1.0421	−1.4396
C	0.1210	−2.2274	−0.7021
C	0.1210	−2.2274	0.7021
C	0.0249	−1.0421	1.4396
C	0.0206	0.2197	0.7375
C	0.0206	0.2197	−0.7375
H	−0.0304	3.6376	1.2497
H	−0.0304	3.6376	−1.2497
H	0.1738	−3.1798	−1.2386
H	0.1738	−3.1798	1.2386
S	−0.2703	−1.3642	−3.1797
H	0.2675	−0.2454	−3.6963
O	0.1394	1.5242	−2.7898
H	0.1680	2.4547	−3.0579
O	0.1394	1.5242	2.7898
H	0.1680	2.4547	3.0579
S	−0.2703	−1.3642	3.1797
H	0.2675	−0.2454	3.6963

$O{:}HS_{14}^{np}$

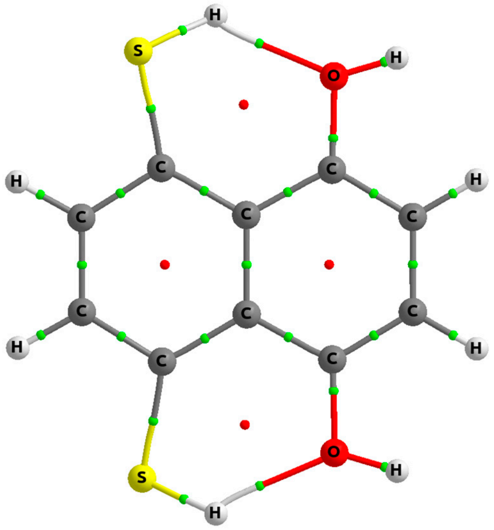

C	−0.0855	−1.4052	1.4899
C	−0.0509	−0.7023	2.6877
C	0.0509	0.7023	2.6877
C	0.0855	1.4052	1.4899
C	0.0013	1.4407	−1.0432
C	0.0026	0.7016	−2.2311
C	−0.0026	−0.7016	−2.2311
C	−0.0013	−1.4407	−1.0432
C	−0.0150	−0.7373	0.2183
C	0.0150	0.7373	0.2183
H	−0.1038	−1.2462	3.6371
H	0.1038	1.2462	3.6371
H	−0.0006	1.2366	−3.1859
H	0.0006	−1.2366	−3.1859
S	−0.2040	3.1955	−1.3604
H	0.3502	3.6790	−0.2353
O	0.2207	2.7846	1.5222
H	0.2546	3.0531	2.4525
O	−0.2207	−2.7846	1.5222
H	−0.2546	−3.0531	2.4525
S	0.2040	−3.1955	−1.3604
H	−0.3502	−3.6790	−0.2353

$O{:}HS_{15}^{p}$

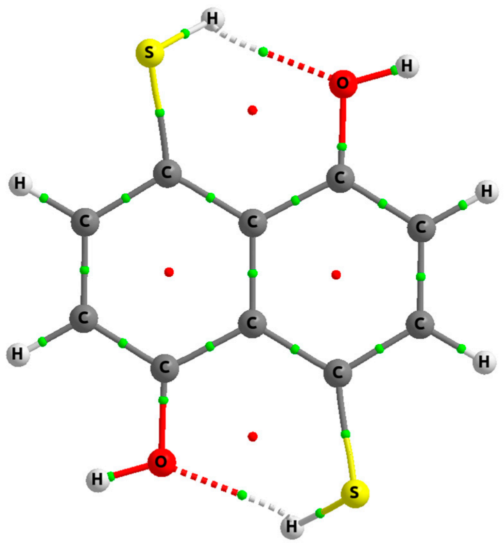

C	1.8005	0.6650	0.0389
C	1.5471	2.0392	0.0137
C	0.2347	2.5446	−0.0326
C	−0.8572	1.6835	−0.0411
C	−1.8003	−0.6649	0.0389
C	−1.5472	−2.0391	0.0137
C	−0.2347	−2.5445	−0.0327
C	0.8572	−1.6835	−0.0412
C	0.6888	−0.2581	0.0083
C	−0.6886	0.2582	0.0084
H	2.3925	2.7326	0.0222
H	0.0680	3.6268	−0.0798
H	−2.3926	−2.7325	0.0221
H	−0.0681	−3.6267	−0.0800
S	−3.5369	−0.2974	0.3393
H	−3.6816	0.7365	−0.5108
O	−2.1331	2.2006	−0.1402
H	−2.0545	3.1666	−0.1554
S	3.5370	0.2974	0.3394
H	3.6814	−0.7372	−0.5099
O	2.1331	−2.2005	−0.1406
H	2.0546	−3.1665	−0.1558

$O{:}HS_{15}^{np}$

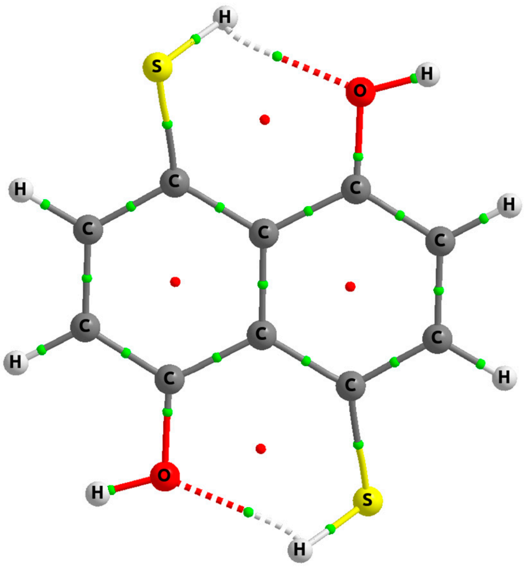

C	1.7925	0.6852	0.0418
C	1.5209	2.0565	0.0668
C	0.2037	2.5469	−0.0027
C	−0.8753	1.6729	−0.0836
C	−1.7925	−0.6852	−0.0418
C	−1.5209	−2.0565	−0.0668
C	−0.2037	−2.5469	0.0027
C	0.8753	−1.6729	0.0836
C	0.6918	−0.2508	0.0225
C	−0.6918	0.2508	−0.0225
H	2.3571	2.7598	0.1108
H	0.0247	3.6282	−0.0207
H	−2.3571	−2.7598	−0.1108
H	−0.0247	−3.6282	0.0207
S	−3.5399	−0.3415	0.2139
H	−3.6746	0.7004	−0.6281
O	−2.1502	2.1747	−0.2504
H	−2.0799	3.1408	−0.2846
S	3.5399	0.3415	−0.2139
H	3.6746	−0.7004	0.6281
O	2.1502	−2.1747	0.2504
H	2.0799	−3.1408	0.2846

O:HSe14p	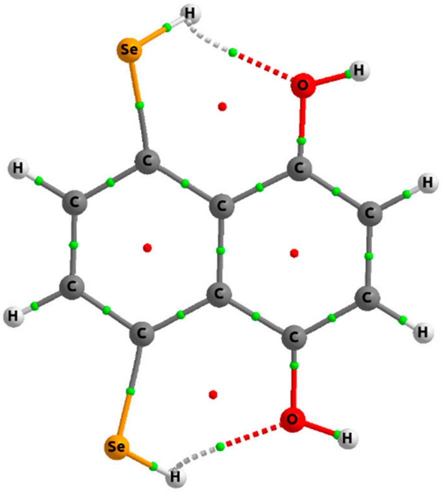	C	−1.5144	−0.0101	1.4104
		C	−2.7094	−0.0952	0.7043
		C	−2.7094	−0.0952	−0.7043
		C	−1.5144	−0.0101	−1.4104
		C	1.0120	0.0655	−1.4392
		C	2.1974	0.1641	−0.7042
		C	2.1974	0.1641	0.7042
		C	1.0120	0.0655	1.4392
		C	−0.2472	0.0224	0.7361
		C	−0.2472	0.0224	−0.7361
		H	−3.6591	−0.1293	1.2494
		H	−3.6591	−0.1293	−1.2494
		H	3.1495	0.2373	−1.2381
		H	3.1495	0.2373	1.2381
		H	0.3830	0.6860	−3.8697
		O	−1.5348	0.0774	−2.7892
		H	−2.4604	0.0234	−3.0711
		O	−1.5348	0.0774	2.7892
		H	−2.4604	0.0234	3.0711
		H	0.3830	0.6860	3.8697
		Se	1.3632	−0.2469	−3.3149
		Se	1.3632	−0.2469	3.3149
O:HSe14np	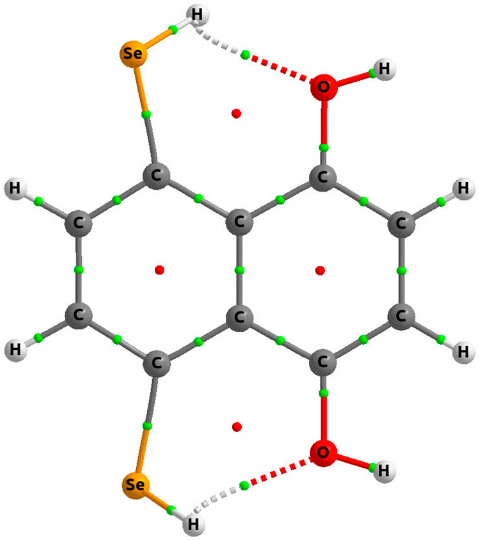	C	0.0031	1.4105	1.5148
		C	0.0087	0.7043	2.7123
		C	−0.0087	−0.7043	2.7123
		C	−0.0031	−1.4105	1.5148
		C	0.0499	−1.4386	−1.0130
		C	0.0218	−0.7038	−2.2018
		C	−0.0218	0.7038	−2.2018
		C	−0.0499	1.4386	−1.0130
		C	−0.0209	0.7354	0.2472
		C	0.0209	−0.7354	0.2472
		H	0.0283	1.2496	3.6622
		H	−0.0283	−1.2496	3.6622
		H	0.0353	−1.2368	−3.1572
		H	−0.0353	1.2368	−3.1572
		H	−0.6037	−3.8666	−0.4167
		O	−0.0482	−2.7913	1.5306
		H	−0.0142	−3.0734	2.4571
		O	0.0482	2.7913	1.5306
		H	0.0142	3.0734	2.4571
		H	0.6037	3.8666	−0.4167
		Se	−0.3803	3.3155	−1.3478
		Se	0.3803	−3.3155	−1.3478

$O{:}HSe_{15}^{p}$	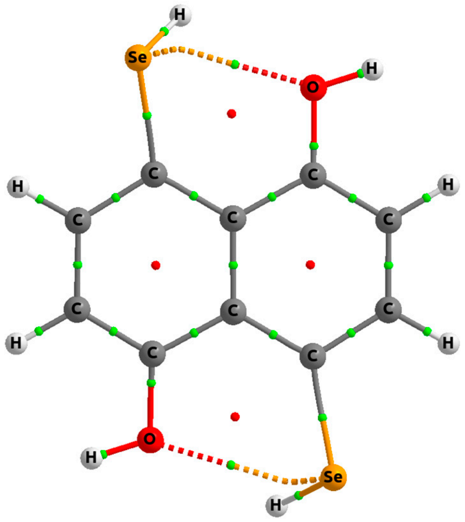	C	−1.7885	0.6934	−0.0008
		C	−1.5114	2.0635	0.0218
		C	−0.1902	2.5465	0.0895
		C	0.8864	1.6660	0.1022
		C	1.7886	−0.6934	−0.0006
		C	1.5114	−2.0636	0.0220
		C	0.1901	−2.5467	0.0896
		C	−0.8863	−1.6660	0.1023
		C	−0.6922	−0.2450	0.0387
		C	0.6923	0.2450	0.0388
		H	−2.3418	2.7745	0.0018
		H	−0.0056	3.6254	0.1472
		H	2.3418	−2.7746	0.0020
		H	0.0054	−3.6255	0.1473
		H	3.9085	0.5915	0.7301
		O	2.1700	2.1467	0.2227
		H	2.1216	3.1151	0.2248
		H	−3.9084	−0.5914	0.7299
		O	−2.1700	−2.1467	0.2228
		H	−2.1216	−3.1150	0.2250
		Se	−3.6543	0.3339	−0.3762
		Se	3.6544	−0.3340	−0.3760
$O{:}HSe_{15}^{np}$	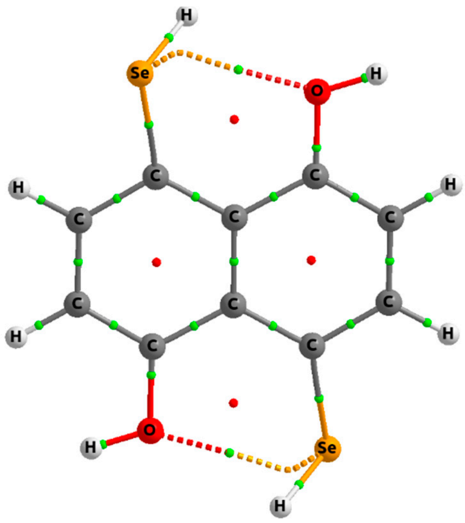	C	1.8033	0.6556	0.0114
		C	1.5536	2.0301	0.0802
		C	0.2419	2.5419	0.0698
		C	−0.8520	1.6855	−0.0044
		C	−1.8032	−0.6555	−0.0112
		C	−1.5536	−2.0300	−0.0801
		C	−0.2419	−2.5419	−0.0699
		C	0.8520	−1.6856	0.0042
		C	0.6876	−0.2602	−0.0058
		C	−0.6875	0.2602	0.0058
		H	2.3988	2.7226	0.1196
		H	0.0787	3.6256	0.0885
		H	−2.3989	−2.7224	−0.1193
		H	−0.0788	−3.6256	−0.0888
		H	−3.8826	0.7143	−0.7046
		O	−2.1239	2.1986	−0.1148
		H	−2.0525	3.1653	−0.0951
		H	3.8823	−0.7145	0.7052
		O	2.1239	−2.1987	0.1144
		H	2.0525	−3.1654	0.0945
		Se	−3.6666	−0.2768	0.3513
		Se	3.6668	0.2767	−0.3507
IMYB[2]					

O:O	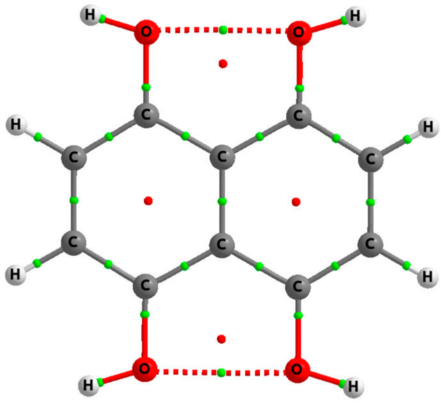	C	0.0000	1.2600	1.4209
		C	0.0000	2.4539	0.7063
		C	0.0000	2.4539	−0.7063
		C	0.0000	1.2600	−1.4209
		C	0.0000	−1.2600	−1.4209
		C	0.0000	−2.4539	−0.7063
		C	0.0000	−2.4539	0.7063
		C	0.0000	−1.2600	1.4209
		C	0.0000	0.0000	0.7315
		C	0.0000	0.0000	−0.7315
		H	0.0000	3.4064	1.2480
		H	0.0000	3.4064	−1.2480
		H	0.0000	−3.4064	−1.2480
		H	0.0000	−3.4064	1.2480
		O	0.0000	−1.2626	−2.8012
		H	0.0000	−2.1899	−3.0822
		O	0.0000	1.2626	−2.8012
		H	0.0000	2.1899	−3.0822
		O	0.0000	−1.2626	2.8012
		H	0.0000	−2.1899	3.0822
		O	0.0000	1.2626	2.8012
		H	0.0000	2.1899	3.0822
O:S_{14}	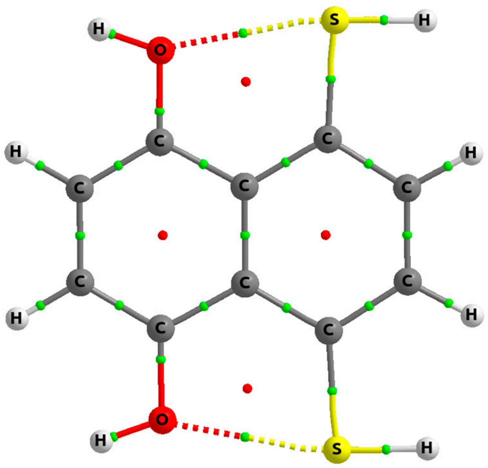	C	0.0396	1.4059	1.5041
		C	0.0199	0.7060	2.7041
		C	−0.0199	−0.7060	2.7041
		C	−0.0396	−1.4059	1.5041
		C	−0.0407	−1.4452	−1.0139
		C	−0.0198	−0.7029	−2.1996
		C	0.0198	0.7029	−2.1996
		C	0.0407	1.4452	−1.0139
		C	0.0206	0.7320	0.2374
		C	−0.0206	−0.7320	0.2374
		H	0.0352	1.2532	3.6527
		H	−0.0352	−1.2532	3.6527
		H	−0.0345	−1.2206	−3.1637
		H	0.0345	1.2206	−3.1637
		S	−0.0913	−3.2372	−1.1308
		H	−0.0910	−3.2239	−2.4836
		O	−0.0784	−2.7856	1.4841
		H	−0.0875	−3.1053	2.3983
		O	0.0784	2.7856	1.4841
		H	0.0875	3.1053	2.3983
		S	0.0913	3.2372	−1.1308
		H	0.0910	3.2239	−2.4836

O:S15

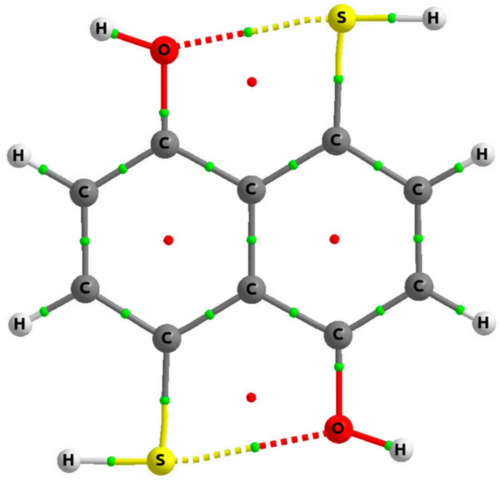

C	1.8288	0.5819	0.0272
C	1.6296	1.9657	0.0297
C	0.3373	2.5271	0.0140
C	−0.7889	1.7120	−0.0045
C	−1.8288	−0.5819	−0.0272
C	−1.6296	−1.9657	−0.0297
C	−0.3373	−2.5271	−0.0140
C	0.7889	−1.7120	0.0045
C	0.6749	−0.2848	0.0082
C	−0.6749	0.2848	−0.0082
H	2.4915	2.6390	0.0441
H	0.2180	3.6163	0.0164
H	−2.4915	−2.6390	−0.0441
H	−0.2180	−3.6163	−0.0164
S	−3.5204	0.0337	−0.0482
H	−4.0527	−1.2097	−0.0602
O	−2.0566	2.2505	−0.0200
H	−1.9801	3.2161	−0.0137
S	3.5204	−0.0337	0.0482
H	4.0527	1.2097	0.0602
O	2.0566	−2.2505	0.0200
H	1.9801	−3.2161	0.0137

O:Se14

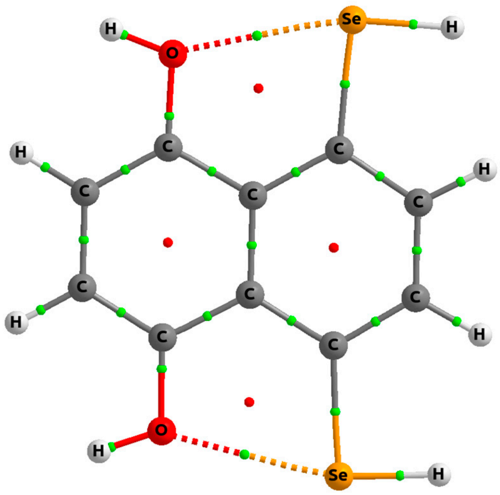

C	0.0371	1.4037	1.5188
C	0.0186	0.7066	2.7201
C	−0.0186	−0.7066	2.7201
C	−0.0371	−1.4037	1.5188
C	−0.0383	−1.4450	−0.9958
C	−0.0186	−0.7035	−2.1823
C	0.0186	0.7035	−2.1823
C	0.0383	1.4450	−0.9958
C	0.0194	0.7311	0.2526
C	−0.0194	−0.7311	0.2526
H	0.0331	1.2540	3.6686
H	−0.0331	−1.2540	3.6686
H	−0.0323	−1.2191	−3.1472
H	0.0323	1.2191	−3.1472
Se	−0.0895	−3.3695	−1.1340
H	−0.0881	−3.3013	−2.6047
O	−0.0734	−2.7842	1.4913
H	−0.0819	−3.1138	2.4019
O	0.0734	2.7842	1.4913
H	0.0819	3.1138	2.4019
Se	0.0895	3.3695	−1.1340
H	0.0881	3.3013	−2.6047

O:Se15

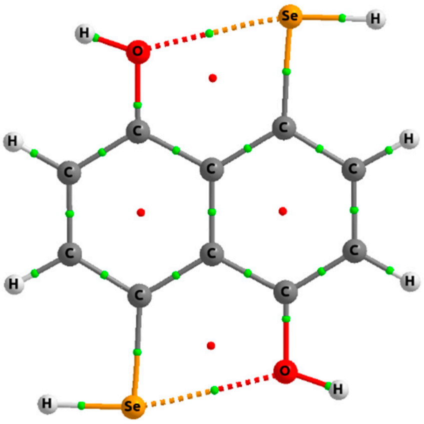

C	1.8285	0.5772	0.0262
C	1.6339	1.9620	0.0293
C	0.3414	2.5259	0.0144
C	−0.7843	1.7110	−0.0035
C	−1.8285	−0.5772	−0.0262
C	−1.6339	−1.9620	−0.0293
C	−0.3414	−2.5259	−0.0144
C	0.7843	−1.7110	0.0035
C	0.6740	−0.2853	0.0078
C	−0.6740	0.2853	−0.0078
H	2.4950	2.6354	0.0431
H	0.2231	3.6154	0.0173
H	−2.4950	−2.6354	−0.0431
H	−0.2231	−3.6154	−0.0173

Se	−3.6500	0.0740	−0.0483
H	−4.1815	−1.2986	−0.0601
O	−2.0548	2.2447	−0.0180
H	−1.9870	3.2110	−0.0134
Se	3.6500	−0.0740	0.0483
H	4.1815	1.2986	0.0601
O	2.0548	−2.2447	0.0180
H	1.9870	−3.2110	0.0134

Figure S2. Molecular graphs and Cartesian coordinates corresponding to those systems with two simultaneous intramolecular interactions at the MP2/aug−cc−pVDZ computational level.
